# Feasibility of screening for diabetic retinopathy using artificial intelligence, Brazil

**DOI:** 10.2471/BLT.22.288580

**Published:** 2022-08-22

**Authors:** Fernando Korn Malerbi, Gustavo Barreto Melo

**Affiliations:** a Department of Ophthalmology and Visual Sciences, Federal University of São Paulo, R. Botucatu 822, 04023-062 São Paulo, SP Brazil.

## Abstract

**Problem:**

There is currently no national strategy or standardized approach to diabetic retinopathy screening in the Brazilian public health system, and multiple socioeconomic barriers prevent access to eye examination in Brazil’s poorest regions.

**Approach:**

From September 2021 to March 2022 we carried out a pilot project with an artificial intelligence system for diabetic retinopathy screening, embedded in a portable retinal camera. Patients with a diagnosis of diabetes according to the municipality registry were invited to attend nearby clinics for screening on designated days. Trained health-care technicians acquired images which were automatically evaluated by the system, with instant remote evaluation by retinal specialists in selected cases.

**Local setting:**

Our study was based in Sergipe State, located at a region with high illiteracy rates and no local availability of specialized retina care. The average number of laser treatments performed annually in the last 5 years is 126, for a total State population of 2.3 million.

**Relevant changes:**

Even though screening was performed free of charge in a convenient location for patients, from a total 2052 eligible individuals, only 1083 attended for screening.

**Lessons learnt:**

Efforts to raise awareness on the condition screened and to provide health education for patients and local health-care personnel are fundamental for increased attendance. Tailoring screening systems to the local setting, such as determining the trade-off between sensitivity and specificity, is challenging in regions with no current benchmarks. Standards for retinopathy screening based on the strategies adopted by high-income countries may not be realistic in low- and middle-income countries.

## Résumé

**Problème:**

Le système de santé publique brésilien ne possède à ce jour ni stratégie nationale, ni approche standardisée pour le dépistage de la rétinopathie diabétique, et de nombreux obstacles socioéconomiques entravent l'accès à des examens ophtalmologiques dans les régions les plus défavorisées du Brésil.

**Approche:**

Entre septembre 2021 et mars 2022, nous avons mené un projet pilote où l'intelligence artificielle était intégrée dans une caméra rétinienne portable afin de dépister la rétinopathie diabétique. Les patients souffrant de diabète, selon le registre de la municipalité, ont été invités à se rendre dans les cliniques des environs pour un dépistage lors de journées prévues à cet effet. Les images réalisées par des techniciens de santé qualifiés ont été automatiquement évaluées par le dispositif et, dans certains cas, soumises à des spécialistes de la rétine pour examen.

**Environnement local:**

Notre étude s'est déroulée dans l'État de Sergipe, une région où le taux d'analphabétisme est élevé et qui ne dispose d'aucun établissement proposant une prise en charge des maladies rétiniennes. Sur une base annuelle, 126 traitements au laser ont été effectués en moyenne ces cinq dernières années, alors que l'État compte 2,3 millions d'habitants.

**Changements significatifs:**

Malgré la gratuité du dépistage et la proximité géographique avec les patients, ils n'ont été que 1083 à venir sur les 2052 individus éligibles au total.

**Leçons tirées:**

Des efforts sont nécessaires pour sensibiliser au dépistage de cette maladie et offrir une éducation sanitaire aux patients et aux soignants de la région, afin de garantir un meilleur taux de fréquentation. Adapter les systèmes de dépistage au contexte local, notamment en trouvant le juste milieu entre sensibilité et spécificité, représente un défi dans des régions actuellement sans repères. Les normes de dépistage de la rétinopathie diabétique inspirées des stratégies en vigueur dans les pays à revenu élevé pourraient se révéler irréalisables dans les pays à revenu faible et intermédiaire.

## Resumen

**Situación:**

En la actualidad, no existe una estrategia nacional ni un enfoque estandarizado para el cribado de la retinopatía diabética en el sistema sanitario público brasileño, y múltiples barreras socioeconómicas impiden el acceso a la exploración oftalmológica en las regiones más pobres de Brasil.

**Enfoque:**

De septiembre de 2021 a marzo de 2022, se llevó a cabo un proyecto piloto con un sistema de inteligencia artificial para el cribado de la retinopatía diabética, integrado en una cámara de retina portátil. Se invitó a los pacientes con diagnóstico de diabetes según el registro del municipio a acudir a las clínicas cercanas para el cribado en días determinados. Los técnicos sanitarios capacitados adquirieron imágenes que el sistema evaluó de manera automática, y los especialistas en retina realizaron una evaluación instantánea a distancia en casos seleccionados.

**Marco regional:**

Este estudio se realizó en el estado de Sergipe, ubicado en una región con altas tasas de analfabetismo y sin disponibilidad local de atención especializada en retina. El promedio de tratamientos con láser realizados al año en los últimos 5 años es de 126, para una población total del estado de 2,3 millones.

**Cambios importantes:**

A pesar de que el cribado se realizó de forma gratuita en un lugar conveniente para los pacientes, de un total de 2052 personas que cumplían los requisitos, solo 1083 acudieron al cribado.

**Lecciones aprendidas:**

Para aumentar la asistencia, es fundamental realizar esfuerzos de sensibilización sobre la enfermedad que se estudia y proporcionar educación sanitaria a los pacientes y al personal sanitario local. La adaptación de los sistemas de cribado al entorno local, como la determinación del equilibrio entre la sensibilidad y la especificidad, es un reto en las regiones que carecen de puntos de referencia actuales. Los estándares para el cribado de la retinopatía a partir de las estrategias que adoptan los países de ingresos altos pueden no ser realistas en los países de ingresos bajos y medios.

## ملخص

**المشكلة:**

لا توجد حاليًا استراتيجية وطنية أو أسلوب قياسي لفحص اعتلال الشبكية السكري في نظام الصحة العامة البرازيلي، وهناك العديد من الحواجز الاجتماعية والاقتصادية التي تمنع الخضوع لفحص العين في المناطق الأكثر فقرًا في البرازيل.

**الأسلوب:**

من سبتمبر/أيلول 2021 إلى مارس/آذار 2022، قمنا بتنفيذ مشروع تجريبي باستخدام نظام ذكاء اصطناعي لفحص اعتلال الشبكية السكري، مدمج في كاميرا شبكية محمولة. المرضى الذين تم تشخيص إصابتهم بمرض السكري وفقًا لسجل البلدية، تمت دعوتهم للحضور للعيادات القريبة للخضوع للفحص في أيام مقررة. حصل فنيو الرعاية الصحية المدربون على صور تم تقييمها أوتوماتيكيًا بواسطة النظام، مع تقييم فوري عن بُعد بواسطة متخصصين في شبكية العين في حالات مختارة.

**المواقع المحلية:**

اتخذت دراستنا من ولاية سيرغيبي مركزًا لها، والواقعة في منطقة ترتفع فيها معدلات الأمية ولا تتوفر رعاية محلية متخصصة لشبكية العين. متوسط عدد العلاجات بالليزر التي تم إجراؤها سنويًا في آخر 5 سنوات هو 126، لإجمالي عدد سكان الولاية البالغ 2.3 مليون نسمة.

**التغيّرات ذات الصلة:**

على الرغم من إجراء الفحص مجانًا في مكان مناسب للمرضى، فمن إجمالي 2052 فردًا مؤهلاً، حضر 1083 فردًا فقط للفحص.

**الدروس المستفادة:**

إن الجهود المبذولة لزيادة الوعي بالحالة التي يتم فحصها، وتوفير التوعية الصحية للمرضى وموظفي الرعاية الصحية المحليين، تعتبر مطلبًا أساسيًا لزيادة الحضور. إن تصميم أنظمة الفحص وفقًا للمواقع المحلية، مثل تحديد المقايضة بين الحساسية والنوعية، يمثل تحديًا صعبًا في المناطق التي لا توجد بها معايير حالية. إن معايير فحص اعتلال الشبكية القائمة على الاستراتيجيات المعتمدة في الدول ذات الدخل المرتفع، قد لا تكون واقعية في الدول ذات الدخل المنخفض والدخل المتوسط.

## 摘要

**问题:**

巴西公共卫生系统目前没有针对糖尿病视网膜病变筛查的国家战略或标准化方法，而且多种社会经济障碍阻碍了巴西最贫困地区进行眼科检查。

**方法:**

从 2021 年 9 月到 2022 年 3 月，我们开展了一个试点项目，将人工智能系统嵌入便携式视网膜摄像头，以便进行糖尿病视网膜病变筛查。市政登记处中诊断为糖尿病的患者被邀请在指定日期到附近的诊所参加筛查。训练有素的医疗技术人员获取系统自动评估的图像，视网膜专科医生对所选病例进行即时远程评估。

**当地状况:**

我们研究的对象是塞尔希培州，该州文盲率高，当地没有专门的视网膜护理服务。该州总人口 230 万，但在过去 5 年中，每年进行激光治疗的平均次数为 126 次。

**相关变化:**

尽管是在便于患者前往的地点免费进行筛查，但在 2052 名符合筛查条件的人中，只有 1083 人参加了筛查。

**经验教训:**

应努力提高人们对筛查疾病的认识，并为患者和当地卫生保健人员提供健康教育，这是提高筛查率的基础。结合当地状况量身制定筛查系统，例如，如何在当前没有基准的地区对敏感性和特异性进行权衡，这便是一项挑战。将高收入国家采用的视网膜筛查战略作为标准，对于中低收入国家来说可能并不实际。

## Резюме

**Проблема:**

В настоящее время в системе общественного здравоохранения Бразилии не существует национальной стратегии или стандартизированного подхода к скринингу на диабетическую ретинопатию, а многочисленные социально-экономические барьеры препятствуют доступу к офтальмологическому обследованию в беднейших регионах Бразилии.

**Подход:**

С сентября 2021 г. по март 2022 г. авторы реализовали экспериментальный проект с использованием системы искусственного интеллекта для скрининга на диабетическую ретинопатию, которая была встроена в портативную ретинальную камеру. Пациентов с диагнозом «диабет», чьи соответствующие данные были упомянуты в муниципальном реестре, приглашали посетить близлежащие клиники для проведения скрининга в назначенные дни. Обученные технические работники здравоохранения получали изображения, которые система автоматически оценивала, а в отдельных случаях ретинологи проводили мгновенную дистанционную оценку.

**Местные условия:**

Данное исследование проводилось в штате Сержипи, расположенном в регионе с высоким уровнем неграмотности и отсутствием специализированной офтальмологической помощи на местном уровне. Ежегодно за последние 5 лет здесь выполнялось в среднем 126 лазерных офтальмологических процедур при общей численности населения штата в 2,3 миллиона человек.

**Осуществленные перемены:**

Несмотря на то что скрининг проводился бесплатно в удобном для пациентов месте, из 2052 человек, соответствующих критериям, только 1083 человека посетили скрининг.

**Выводы:**

Усилия, направленные на повышение осведомленности о подлежащем скринингу заболевании и медико-санитарное просвещение пациентов и местного медицинского персонала, имеют основополагающее значение для повышения посещаемости. Адаптация систем скрининга к местным условиям, например определение компромисса между чувствительностью и специфичностью, является сложной задачей в регионах, где нет действующих контрольных показателей. Стандарты скрининга на ретинопатию, основанные на стратегиях, принятых в странах с высоким уровнем дохода, могут оказаться нереалистичными в странах с низким и средним уровнем дохода.

## Introduction

Major public health issues need scalable solutions, especially in low- and middle-income countries where the health workforce is scarce and public health systems are under-resourced.^1^ Recently, reports of implementation of artificial intelligence systems have been published, and the screening of diabetic retinopathy, one of the leading causes of preventable blindness in the adult working population,^2^ is one such example. Estimates show that there will be more than half a billion persons with diabetes by 2030.^3^ Given that each patient needs at least one annual retinal examination, the traditional individualized assessment by retinal specialists may not be sustainable in all health-care systems. 

A recent study in Thailand showed the feasibility of using artificial intelligence for diabetic retinopathy screening in a national strategy;^4^ favourable results were found as compared with the regional standard of care. The authors emphasized the importance of socioenvironmental factors and screening workflows for successful implementation. However, in some low- and middle-income countries, there are no official validated solutions or established standards of care for several important public health conditions. This issue poses the question: what are the benchmarks against which an artificial intelligence-based strategy should be compared? In many cases, standards based on the strategies adopted by high-income countries may not be realistic in low- and middle-income countries.

We share our experience of diabetic retinopathy screening that may provide useful lessons for other low- and middle-income countries.

## Local setting

Brazil is a large upper-middle-income country that hosts the sixth largest population of individuals with diabetes in the world.^5^ The country also has the largest free public health-care system,^6^ on which the majority of its population relies. There are currently no national strategies or standardized workflows for diabetic retinopathy screening in the Brazilian public health system. Multiple socioeconomic barriers prevent access to eye examination in the poorest regions, as is the case of the remote areas of the north-eastern state of Sergipe.^7^ Sergipe State is located in a region with high illiteracy rates and no availability of specialized retinal care outside of the State’s capital.^8^ According to available data on the official database of Brazil’s public health system, the average number of laser treatments performed annually in the last 5 years was 126, for a total State population of 2.3 million. 

## Approach

From September 2021 to March 2022 we did a pilot project in four rural villages that have no available ophthalmology care, in Sergipe State’s drylands (total population 76 000; human development index (HDI) range: 0.529–0.602).^7^ Patients with a diagnosis of diabetes according to the municipality registry were invited by local health agents to attend for screening on designated days. 

The screening consisted of retinal imaging with a previously validated, highly sensitive, offline artificial intelligence system, embedded in a portable retinal camera.^8^ Image acquisition was performed by trained health-care technicians, with automatic evaluation by the system, and instant remote evaluation by retinal specialists in selected cases. Screening was performed in primary-care clinics located close to the patients’ place of residence. Due to the system’s high sensitivity, only patients identified as suitable for referral (more than mild diabetic retinopathy or images with poor quality) were sent for remote evaluation by retinal specialists, the technician being instantly notified on the need for referral. Laser treatment was offered free of charge to all individuals with vision-threatening diabetic retinopathy detected. 

## Relevant changes

Although screening was performed free of charge in a convenient location for patients, from a total 2052 eligible individuals, only 1083 attended the screening. Since there is no legal framework for artificial intelligence in health in Brazil ‒ such discussion is currently underway in the Brazilian Senate ‒ all examinations were eventually reassessed by specialists afterwards; no vision-threatening case was missed.

## Lessons learnt

First, although screening provided by artificial intelligence systems at the point of care correlates with improved adherence to referrals,^1^^,^^4^ we believe the lack of awareness about diseases, complications and prevention among the public is a major issue accounting for a low attendance in our setting. Even in countries with well-established screening programmes, only approximately 18% to 60% of individuals undergo examinations.^3^ Continuous education of patients, and health-care providers and authorities, is important for the prevention of chronic complications of diabetes.^9^ Comprehensive efforts and communication campaigns should therefore be made to engage the whole society and to increase awareness and adherence rates (Box 1).^8^ Additionally, we scheduled home visits for patients unable to go to the examination site whenever possible. Furthermore, it should be noted that this kind of screening has never been performed in this region. We believe that continuity of the programme would allow patients who missed one opportunity for examination to attend subsequent screenings.

Box 1 **Summary of main lessons learnt**

- Raising awareness about diabetic retinopathy among patients and local health-care workers is essential for increased attendance and the overall success of screening initiatives.
- Tailoring screening systems to the local setting, such as determining the trade-off between sensitivity and specificity, is challenging in regions with no current benchmarks.
- Cost considerations are essential for the sustainability of diabetic retinopathy programmes; health outcome metrics should be sought to evaluate the impact of such programmes.

Second, the design of the strategy should be tailored to account for existing or proposed workflows. To reduce the risk of ungradable images, the training of personnel on the acquisition of ocular images should be emphasized and measured.^6^ Operators could also be trained for image quality assessment, allowing the implementation of a staged mydriasis strategy. A protocol which also includes anterior segment images allows for identification of cataracts, which may also be considered referral criteria, as lens opacities are a frequent cause of visual impairment in such patients.^6^

Third, cost decisions have to be customized to each setting to fit local resource constraints. These decisions could include the predefined end-points for sensitivity and specificity; the threshold for referral; the criteria for considering an image as gradable or ungradable; autonomous versus semi-autonomous workflows; systems that detect multiple diseases versus disease-specific systems; and the use of other variables such as visual acuity.^4^ When defining or calibrating the screening system’s cut-off, choices are made in the form of a trade-off between sensitivity and specificity. As an example, the Exeter protocol, put forward in 1995, proposed a minimum sensitivity of 80% and a minimum specificity of 95% for the screening of vision-threatening diabetic retinopathy. With annual screenings, this level of sensitivity could be adequate because repeated examinations tend to detect retinopathy missed at earlier examinations.^10^ Since referrals incur costs for the patient and the health-care system,^4^ the emphasis on specificity over sensitivity has been a benchmark for several diabetic retinopathy screening initiatives. In our example, the artificial intelligence system had been previously optimized from a screening perspective for a high sensitivity to yield fewer false negatives. We could have used a semi-automated workflow that maintained expert human reading only for cases identified as referable, provided the system was approved by the national regulatory agency. Such an approach has been reported to be cost-effective.^11^

Many other challenges will arise in the deployment of artificial intelligence systems, such as the absence of universal electronic records, slow internet speeds, operator-related and camera-related technical issues, and the lack of standardization for the exchange of retinal images and clinical data.^12^ In the example from Thailand mentioned above,^4^ the reported advantages of advanced technology for diabetic retinopathy screening are not yet proven through formal cost‒benefit and cost‒utility analyses^1^^,^^2^ However, the recent implementation of artificial intelligence for diabetic retinopathy screening has also been reported in other low- and middle-income countries such as India and Rwanda.^1^^,^^13^ In India, several studies reported high diagnostic accuracy of artificial intelligence for diabetic retinopathy screening and its feasibility in the primary-care setting. In the case of Rwanda, immediate feedback of the screening results to patients was related to better adherence to follow-up appointments. The real success of artificial intelligence systems is related not only to the diagnostic accuracy but also to real improvement in the health of the population.^12^ Further studies are necessary to evaluate such outcomes.

Ensuring subsequent specialized care is important for those patients identified by the screening programme and it is also necessary to consider the ability of the community health-care system to accommodate the additional numbers of patients identified.^3^ Underserved areas already lack sufficient treatment coverage, and the systematic identification of new cases of diabetic retinopathy will certainly increase the demand for treatment in the early phases of the programme. Our initiative included advocacy directed at the local health authorities for the adoption and implementation of a cost-effective model for the screening of diabetic retinopathy, with the aim of attaining financial sustainability for the programme. Other ethical aspects of artificial intelligence,^14^ such as non-maleficence (the expert over-reading of all images not to miss false negatives) and the promotion of equity (diagnosis and treatment were offered to all eligible patients free of charge) were also elements of the initiative.

Finally, incomplete data and the lack of representative data sets in the development of artificial intelligence may lead to bias. Public data sets of diabetic retinopathy images represent samples from only seven countries, leaving most of the global population unrepresented.^15^ Of note, the screening system used in the present initiative was previously validated with a similar sample from a neighbouring state in Brazil.^8^

In conclusion, our experience with an artificial intelligence system for prevention of diabetic blindness highlights challenges that are certainly shared by other low- and middle-income countries. Each country will make choices when deciding on public health screening strategies; decisions will be made in the political sphere, after legislative debates, or following scientific guidelines, for example. We believe that a strategy involving scientific societies or multilateral forums can determine the characteristics of the artificial intelligence system, standardized protocols and key quality metrics. The aim would be to maximize outcomes in a cost-effective and successful manner, given the available resources to ensure the sustainability of such efforts.
